# Cortical Hypoexcitation Defines Neuronal Responses in the Immediate Aftermath of Traumatic Brain Injury

**DOI:** 10.1371/journal.pone.0063454

**Published:** 2013-05-07

**Authors:** Victoria Philippa Anne Johnstone, Edwin Bingbing Yan, Dasuni Sathsara Alwis, Ramesh Rajan

**Affiliations:** Department of Physiology, Monash University, Monash, VIC, Australia; Medical College of Georgia, United States of America

## Abstract

Traumatic brain injury (TBI) from a blow to the head is often associated with complex patterns of brain abnormalities that accompany deficits in cognitive and motor function. Previously we reported that a long-term consequence of TBI, induced with a closed-head injury method modelling human car and sporting accidents, is neuronal hyper-excitation in the rat sensory barrel cortex that receives tactile input from the face whiskers. Hyper-excitation occurred only in supra-granular layers and was stronger to complex than simple stimuli. We now examine changes in the immediate aftermath of TBI induced with same injury method. At 24 hours post-trauma significant sensorimotor deficits were observed and characterisation of the cortical population neuronal responses at that time revealed a depth-dependent suppression of neuronal responses, with reduced responses from supragranular layers through to input layer IV, but not in infragranular layers. In addition, increased spontaneous firing rate was recorded in cortical layers IV and V. We postulate that this early post-injury suppression of cortical processing of sensory input accounts for immediate post-trauma sensory morbidity and sets into train events that resolve into long-term cortical hyper-excitability in upper sensory cortex layers that may account for long-term sensory hyper-sensitivity in humans with TBI.

## Introduction

Traumatic brain injury (TBI) results when an external force damages the brain. It is a major public health concern worldwide since TBI-related injuries have an incidence range of 108–332 per 100 000 of the population per year [1], and are the leading cause of death for persons aged 1–44 years [2]. Development of successful therapies for treatment of these injuries is hindered by the complex neuro-pathophysiology in TBI. In most instances there is dynamic interaction between systemic changes (e.g. hypoxia, hypotension, hypercarbia) and local changes at the injury site, and this interplay is critical in determining patient outcomes [3]. Local changes vary widely, but include glutamatergic excitotoxicity [4]–[6], metabolic perturbations [7], [8], ion imbalance [9], [10], inflammation [11], [12] and oxidative stress [13], [14]. These short-term changes in the cerebral environment appear to induce cortical plasticity and remodelling which can manifest as long-term behavioural derangements [15].

A common feature of many forms of brain injury is cortical neuronal hyper-excitation. This is also true for TBI and cortical hyper-excitation has now been demonstrated in two different models of TBI. In a cortical compression injury model where the injury pulse was delivered directly to the brain exposed through a craniotomy [16], hyper-excitation to paired pulse stimuli was found in the granular layer (the only layer studied) within minutes after injury, and built up over the course of the next 2 hours of experimentation. We used a closed-head injury model [17] which produces the more common type of TBI, diffuse TBI. We found that at 8–10 weeks post-TBI, sensory cortex hyper-excitability to sensory input, but only in supra-granular layers and most prominently to complex stimuli rather than simple ones [18].

The closed head impact injury model we used has very strong face validity [19] with human motor vehicle or sporting field accidents on a number of levels, including that it has the same injury phases as seen from imaging humans in car accidents and some forms of forceful sporting field accidents, it is a closed head injury model and therefore allows for the play of factors that are lost in other common TBI models which create a craniotomy to directly apply injury to the dura (e.g., the cortical compression injury model of Ding *et al*. [16]), and it has strong construct validity in that the impact velocities seen in this model are very comparable to those seen in front-end car-car and car-barrier accidents [20]–[23], for pedestrians in car-pedestrian accidents [24], [25] and in forceful sporting field accidents [26]. Thus it is likely that our model involves the same sort of damage processes as seen in humans sustaining a blow to the head and creates the same sort of damage as occurs to humans under those conditions. Given the direct relevance of our observations of long-term cortical hyper-excitability [18] to the sensory hyper-sensitivity seen in humans with long-term TBI [27]–[29], we have begun to explore when this effect develops in the critical immediate period following TBI as seen in the Ding *et al*. [16] model. Using this model, we have now examined sensory cortical neuronal activity *in vivo* at systematic depths from the cortical surface in the immediate 24 hour period following diffuse TBI. We found significant suppression of neuronal responses to both simple and naturalistic complex patterns of controlled whisker deflections all the way from the upper (supragranular) cortical layers through to input (granular) Layer 4 but not deeper in cortex in Layer 5. There was also an increase in spontaneous activity in Layer 4 and part of Layer 5.

We propose that these early depth-dependent suppressive changes in sensory cortical circuitry are consistent with an impact stress/strain wave radiating from the site of weight impact on the skull and propagating through the underlying brain tissue [17], [30]–[34]. Our previous study [18] indicates that over the course of 8 weeks this depression must resolve to normal responses in granular and infragranular layers but overshoots into hyperexcitability in supragranular layers. Good definition of the time course of these changes will be critical in revealing new strategies for early post-injury clinical intervention to halt deficit progression or the change to long-term hyper-sensitization and related sensory deficits. Better definition of the evolution of cortical changes that appear to be universal across many brain disease conditions may also allow identification of the timing of application of therapeutic agents that have been found beneficial or are being trialled for benefits in other injury-related cortical hyper-excitability conditions.

## Materials and Methods

### Animals

Experiments were performed in accordance with the National Health and Medical Research Council guidelines for the care and welfare of experimental animals, and received approval from the Monash University Standing Committee on Ethics in Animal Experimentation. Male Sprague-Dawley rats (8–12 weeks) were housed under a 12****hr light/dark cycle with food and water *ad libitum* and were trained in behaviour tasks for 2 days before undergoing surgery to create diffuse traumatic brain injury (TBI) or for sham controls. Each animal was randomly allocated to either the TBI or Sham group for surgical treatment, and then kept for 24 hours post-trauma for electrophysiological testing.

### Surgery

Eight rats were treated with the weight-drop impact acceleration method (WDIA) [17] for diffuse TBI, modified as previously described [35] while 6 littermates underwent Sham surgery. All animals were anesthetized with 5% isofluorane via inhalation before being intubated and mechanically ventilated with a maintenance dose of 3.5% isofluorane in 22% oxygen/78% nitrogen. The skull was exposed and a metal disc (1 cm diameter; 3****mm thick) fixed to the skull between bregma and lambda, with dental acrylic. The animal was briefly disconnected from the ventilator, placed on a foam bed under the trauma device and a 450****g weight dropped a distance of 2 m through a vertical tube positioned above the rat’s head, onto the metal disc [17]. Mechanical ventilation was immediately resumed post-trauma with 22% oxygen/78% nitrogen until regular spontaneous breathing was regained. The metal disc was removed, scalp incision was sutured and animals were allowed to recover overnight. Sham animals underwent the same surgical procedure as the TBI animals but did not receive the weight impact. Body temperature was always maintained at 37–38°C, using thermostatically-controlled heating pads during the surgery.

### Behaviour Tests

Sensorimotor function was assessed using the rotarod and beam walk tasks [36]. All animals were trained to perform the tests for 2 days prior to surgery, and re-tested 24 hours post-TBI. The rotarod was used to assess coordination and balance, and required the rat to maintain balance on a rotating rod of increasing speed [37]. The rotational speed increased in increments of 1.5 rpm every 3 seconds, and the highest speed at which the rat was able to maintain its balance on the device was recorded. The rotarod score at 24 hours post-surgery was expressed as a percentage of the pre-surgery score. The beam-walk task assessed the ability to traverse a narrow beam (2 cm wide) suspended between two platforms and was scored as 0 = normal walking for at least 0.5****m; 1 = crawling with abdomen touching the beam; 2 = inability to move on the beam; and 3 = inability to balance on the beam.

### Electrophysiology

Immediately after the behaviour tests at 24 hour post-surgery, electrophysiological recordings from posteromedial barrel subfield (PMBSF; barrel cortex) were obtained using methods previously established in our group [38], [39]. Briefly, animals were anesthetized using 5% halothane and tracheotomised to maintain anaesthesia at 0.5–3.0% halothane through continuous ventilation. Depth of anaesthesia was regularly monitored using ECG/EMG recordings from forepaw musculature, pinch withdrawal reflexes, and palpebral reflexes. Body temperature was maintained between 37–38°C.

A head bar was used to anchor the head in place, and skull over the right barrel cortex (approx. 2****mm caudal to bregma; 6****mm lateral to the midline) was removed via drilling, and the exposed barrel cortex (with dura intact) was penetrated with a tungsten microelectrode (2–4 MOhm; FHC) using a fast-stepping microdrive (Kopf Model 2660) mounted on a complex of translators and goniometers [38], [39].

Manual whisker deflections were carefully applied with a fine probe to accurately determine the Principal Whisker (PW; the whisker providing main excitatory input). Instances in which the electrode was placed in barrel cortex septal regions resulted in weaker responses, less precise responses to movement of the PW, and generally approximately equal responses to movement of multiple whiskers. When this pattern of activity was identified in the first recording point at 600–800 µm from the cortical surface in a penetration, the electrode was withdrawn and moved slightly laterally to make a new penetration. Once responsiveness to a single PW was unequivocally identified, the electrode was moved systematically to different depths in that penetration to record neuronal responses from the various cortical laminae.

To minimise any temporal effects of halothane anaesthesia throughout the experiment, the order in which laminae were recorded from was randomised for each experiment. For example, in some cases we began recording in Layer 5 whereas in others we initially recorded from Layer 2, and then advanced systematically to Layer 5. No time-dependent changes in response characteristics were observed. In addition, we have conducted experiments using this anaesthetic and lasting for over 14 hours, throughout which the responses we recorded very well modelled those seen in awake rats with temporal and spatial responses patterns to vibrissae motion being nearly identical in both states [38], [40], even in the case of recordings from the anaesthesia-vulnerable cerebellum [38]. Output signals from the electrode were amplified and band-pass filtered from 0.3–10 kHz [38], [39]. On-line displays of rasters of spike occurrences and peristimulus time histograms were generated using Spike2 software. A copy of the filtered neural signal was also recorded by Spike2 to allow for offline extraction of single neuron data from the cluster population responses.

### Controlled Whisker Deflections

**Figure 1:**
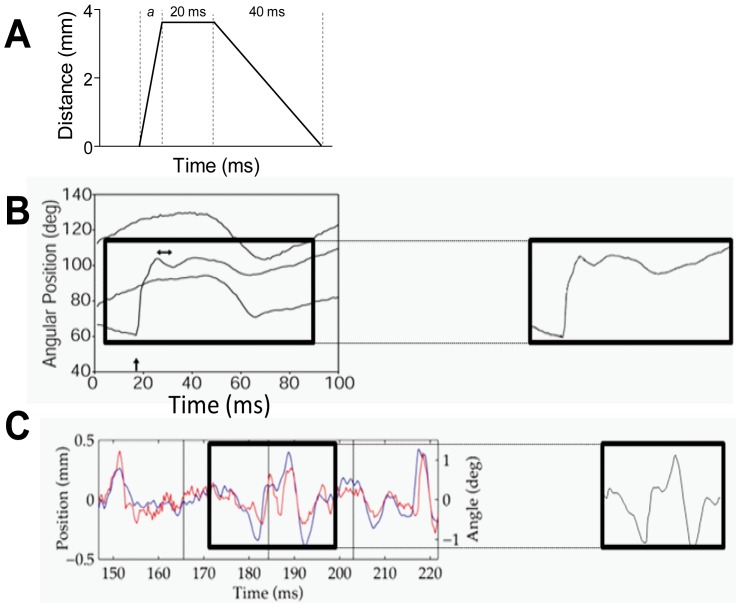
Simple and complex motion waveforms applied to the Principal Whisker of cortical neurons under study. Simple trapezoidal stimuli (**A**) with variable onset ramp velocity (*a*) but fixed amplitude of 3.6 mm, hold duration of 20 ms and offset duration of 40 ms were used. Shown here is the trapezoid with maximum onset ramp velocity of 400 mm/s. (**B**) Object contact whisker motion pattern, recorded by [41] from an awake behaving rat, moving a whisker to contact an object and brush past it. The left panel shows the waveforms as presented in Fig. 8A of that report [41], and the right hand panel shows the specific waveform we applied to the PW. (**C**) Rough surface discriminatory whisker pattern recorded by Ritt *et al* [42] as the whiskers were moved across a rough surface by an awake behaving rat that had been trained to discriminate between rough and smooth surfaces. The left panel shows the waveforms as presented in Figure 3B of that report [42] and the right hand panel shows the specific waveform we applied to the PW. The red line shows the face-centred angle of motion 5 mm from the face, and the blue line is the simultaneous vibrissae motion through a line scan placed 1 mm from the face surface.

The methods for applying simple and complex motion stimuli to the PW of neurons under study have been described previously [38]. In brief, the PW was threaded through a hole on a motor-controlled lever arm system positioned 5****mm from the face. The lever arm was moved under computer control in well-defined motion patterns (see Figure 1) while neural recordings were obtained from barrel cortex.

At each recording location, the first stimuli were always a suite of 5 trapezoidal stimuli (Figure 1A), used to characterise neuronal cluster responses at that point and to extract single unit waveforms. Only the onset ramp velocity was varied in these stimuli, being 30, 60, 150, 250 or 400 mm/s, with deflection amplitude fixed at 3.6 mm, the trapezoid hold duration kept constant at 20 ms, and the offset ramp duration fixed at 40 ms. The entire suite of 5 stimuli was repeated 100–250 times, with each repetition consisting of the five stimuli being presented pseudorandomly, to obtain 350–700 repetitions of each stimulus, to enable reliable spike sorting online. Standard Spike2 template matching algorithms were applied to generate individual spike waveform templates (between 100–1200 samples being used to generate each template) from the responses and in most cases 3–4 waveforms were obtained at any recording location. These templates were applied at that recording point, to separate the responses of the different presumptive neurons to the trapezoid stimuli and the subsequent complex whisker motion stimuli (see below), for later off-line analysis.

Two complex “naturalistic” whisker deflections were then applied in turn to the PW, modelling the whisker motion videographed in rats making contact with a rod placed in the path of the whiskers (Figure 1B [41]) or the whisker motion across rough surfaces made by rats trained to discriminate between rough and smooth surfaces (Figure 1C [42]). The methods for extracting these stimuli from the original reports [41], [42] and then storing and playing them out from text files which stored stimulus characteristics to cause a complex whisker motion in our system have been detailed in our recent report [18]. Ten stimulus amplitudes were used for each of the two complex whisker motions, beginning with an amplitude of 0.2 mm and then continuing from 0.4–3.6 mm, in 0.4 mm steps. Each stimulus amplitude was presented 50 times in a pseudorandom order.

### Data Analysis

All electrophysiological data are represented as firing rate (Hz) in 1 ms bins over the period from 200 ms prior to stimulus onset until 100 ms post-stimulus offset. The data from the clusters was used for offline analysis to generate population peristimulus time histograms to produce a Grand peristimulus time histogram to show the pattern of population responses within a lamina, in TBI and sham animals. A responsive unit was defined as one with responses significantly greater than spontaneous rate over at least 3 successive stimulus amplitudes of the simple or complex stimuli. The peristimulus time histograms were generated by averaging responses to each stimulus amplitude (for the complex stimulus waveforms) or each stimulus ramp velocity (for the simple trapezoid stimuli) across all presentations of that stimulus. The averages were corrected for spontaneous firing rate, using the 200 ms pre-stimulus firing rate. Then a 5-point weighted moving average was applied to smooth out any noise in the responses, and the data were then averaged across all multi-units to produce a Grand peristimulus time histogram.

While the Grand peristimulus time histogram was used to visualize the overall pattern of responses in a lamina to a stimulus, neuronal response metrics were derived separately from each cluster within a lamina. Thus, the peak firing rate, excitatory area under the curve, latency to peak firing rate, and half-peak width were calculated for each cluster for each stimulus. For the trapezoidal whisker motion stimuli these metrics were calculated separately for each trapezoid defined by a particular onset ramp velocity, and for the complex whisker motion stimuli these metrics were calculated separately for each stimulus amplitude. Specific counting windows were used for each stimulus: for simple trapezoidal and complex object contact stimulus a 5–50 ms window after stimulus onset was used; for the rough texture discrimination stimuli a 5–30 ms counting window was used. These specific counting windows were set to encompass the maximum response over the stimulus presentation period. These metrics were grouped for all clusters assigned to a specific lamina (see Results for laminar allocation by recording depth from the cortical surface) and descriptive and inferential statistics generated from these laminar-specific metrics. Detailed results of the statistical analyses are presented in supplementary tables 1, 2 and 3.

**Table 1:** Summary of effects of TBI on firing parameters for each whisker motion stimulus.

Stimuli	Analysis Window (ms)	Peak firing rate changein onset response	Change in excitatory area under the curve in onset response
Trapezoid	5–50	↓L2, U3, D3, L4*No Δ L5*	↓L2, U3, D3, L4*No Δ L5*
Object contract [41]	5–50	↓L2, U3, D3, L4*No Δ L5*	↓L2, U3, D3, L4*No Δ L5*
Rough surface discrimination [42]	5–30	↓L2, U3, D3, L4*No Δ L5*	↓L2, U3, D3, L4*No Δ L5*

The stimulus waveforms are (from top to bottom) trapezoids, object contact [41] and rough surface discrimination [42]. Column 2 indicates the analysis window over which the firing rate parameters were measured. See Figure 1 for illustration of waveform.

### Immunohistochemistry

To identify axonal damage, we used established immunohistochemical methods of staining for β-amyloid precursor protein (β-APP; cat number 512700 batch 985636A) and neurofilament heavy-chain (NF-H; 200 kDa; cat number 131000 batch 1015311A) [35], [36]. After electrophysiological recordings were complete, animals were deeply anaesthetised with Lethobarb (Sodium pentobarbitone; 300 mg/ml) and cardiac perfusioned with 4% paraformaldehyde. After suitable tissue processing brains were randomly selected from each treatment group and immunohistochemical processing was carried out. Staining was performed on consecutive sections in the area 1.3 mm caudal to bregma using standard procedures with polyclonal β-APP antibody (1∶1000; Invitrogen, CA, USA), and monoclonal NF-H antibody (1∶1000; Invitrogen) overnight at 4°C. Labelled cells were visualized by incubating the tissues with fluorescent-tagged secondary antibodies (1∶200 goat anti-rabbit Alexa 594 [red]; Invitrogen, Carlsbad, CA, and 1∶200 goat anti-rabbit Alexa 488 [green]; Invitrogen). Antigen retrieval was performed for detection of both immunohistochemical stains used here. Stained sections were scanned by ScanScope AT Turbo slide scanner (Aperio, CA, USA) and visualised and analysed by Aperio ImageScope (v11.2.0.780, Aperio, CA, USA).

## Results

### Behavioural Changes at 24 Hours Post-TBI

**Figure 2:**
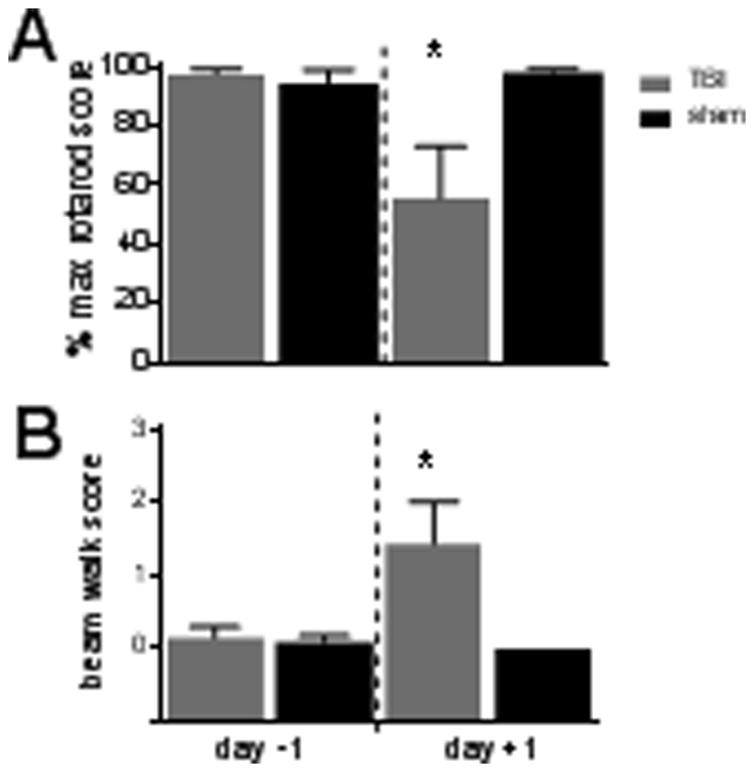
Behavioural deficits following diffuse traumatic brain injury (TBI). Changes in behaviour pre- (day −1) and post- (day +1) surgery for TBI (n = 7) and sham controls (n = 6). Each data point represents data from 1 animal. (**A**) Grip strength and motor co-ordination assessed using the rotarod: Performance for each animal is expressed as % of maximum possible rotarod score. (**B**) Balance and motor co-ordination: beam-walking scores (0 = perfect balance and ability to walk across beam; and 3 = unable to balance on or walk across beam) are displayed for each animal. For all data group mean ± SEM bars are overlayed on top of individual animal data for both TBI (n = 7) and sham (n = 6) controls. (*) *p*<0.05.

Seven of the eight animals subject to treatment with the WDIA method exhibited significant behavioural deficits 24 hours after the treatment. For both the rotarod and beam walk tasks there were no difference in performance between sham and TBI groups pre-trauma, but at 24 hours post-surgery these 7 TBI animals showed a significant decrease in rotarod scores (Fig. 2A, TBI = 54±16%, n = 7; Sham = 98±1%, n = 6; Student’s two-tailed *t* test, *p*<0.05) and in beam walk scores (Fig. 2B, TBI = 1.42±0.57, n = 4; Sham = 0.00±0.00, n = 6; Student’s two-tailed *t* test, *p*<0.05).

One animal treated with the WDIA method did not show any behaviour deficits; the data from this animal are detailed and discussed in the last section of the Results.

### Changes in Neuronal Responses to Simple and Complex Whisker Stimuli Post-TBI

Neuronal responses were obtained 24 hours following injury from the 8 WDIA-treated animals and the 6 sham treated animals, to both simple and complex, “naturalistic”, whisker motion patterns (see Fig. 1). Neuronal data were separated into lamina by depth as Layer 2 (150–300 µm from the cortical surface); Upper Layer 3 (350–500 µm); Deep Layer 3 (550–700 µm); Layer 4 (750–1000 µm); and Layer 5 (1100–1400 µm).

As noted above, one WDIA-treated animal showed no behaviour deficits and so its results are presented separately in the last part of this Results section. For the rest of this Results section, the data from the WDIA-treated animals (the “TBI” animals) consist of group data from the 7 TBI animals showing behaviour deficits 24 hours post-WDIA treatment.

**(a) TBI effects on response strength to variations in whisker protraction velocity:** Whisker protraction velocity is a critical factor in activating barrel cortex neurons, and is frequently studied using simple trapezoidal patterns of whisker motion in which the onset ramp velocity is varied [39], [43]–[45]. We evaluated neuronal responses to this critical whisker motion parameter by using a suite of 5 trapezoidal stimuli with varied onset ramp velocity. Recordings were obtained from a total of 52 responsive multi-unit clusters in sham surgery animals, and 70 responsive multi-unit clusters in TBI animals. There was no significant difference between the two groups in numbers of responsive clusters in each layer (χ^2^ = 1.51, df = 4, *p*>0.05).

**Figure 3:**
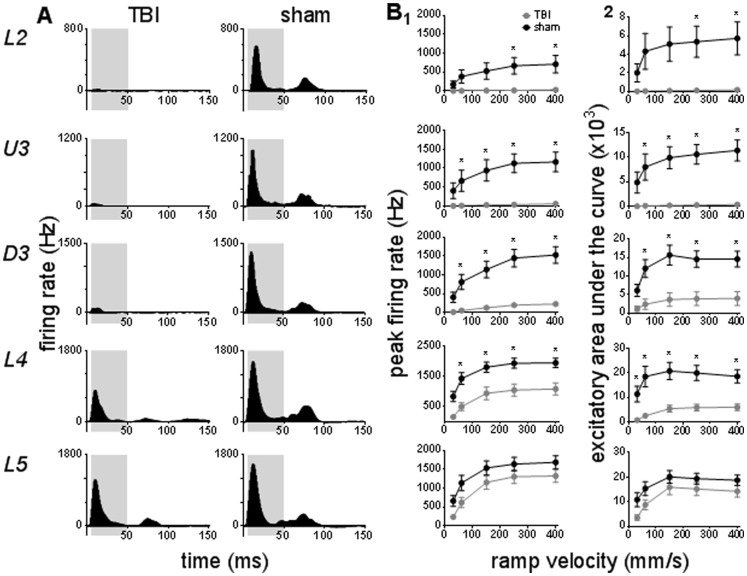
TBI effects on pattern and strength of responses evoked by simple trapezoidal stimuli. Population Grand peristimulus time histograms are shown in (**A**) in response to the trapezoid with the fastest onset ramp velocity (400 mm/sec) in a specific lamina (lamina indicated to left of panel) in TBI and sham animals. Laminar designations: L2 = Layer 2; U3 = Upper Layer 3; D3 = Deep Layer 3; L4 = Layer 4; L5 = Layer 5. Each Grand peristimulus time histogram was generated by averaging responses across all responsive clusters in that lamina. The grey shaded box represents the analysis window used to extract various response onset metrics. (**B**) Peak firing rate (**1**) and excitatory area under the curve (**2**) extracted from the onset response to simple trapezoidal stimuli from clusters in TBI animals (grey circles) and in Sham surgery animals (black circles). Data represents averages from all responsive clusters (±SEM) at all tested ramp velocities, separated by cortical lamina. Each row of data comes from the same lamina as designated by the labels on the left. (*) *p*<0.05.

The Grand peristimulus time histograms obtained at the highest velocity, 400 mm/s, in each of the five laminae in TBI animals and in Sham surgery animals are compared side-by-side in Figure 3A. In sham surgery animals (right column, Fig. 3A), in all layers the population peri-stimulus onset response consisted of a single peak which was followed by low levels of tonic excitation and then a second peak, corresponding to stimulus offset. In direct contrast, in the 7 TBI cases (left column, Fig. 3A) there was a marked suppression of firing rate in all layers such that responses were very poorly defined in Layer 2 and Upper and Deep Layer 3. Responses in Layer 4 and Layer 5 shared similar characteristics to those in sham animals, except with a clear reduction in the amplitude of both onset and offset responses.

To quantify the changes in responses, metrics examining various properties of neuronal responses were extracted from the population data. For the simple trapezoidal stimuli, an analysis window of 5–50 ms from stimulus onset was used to characterise the response to stimulus onset (grey shaded box, Fig. 3A). The response strength was assessed by analysing the peak firing rate (Fig. 3B_1_) and excitatory area under curve (Fig. 3B_2_) during this analysis window. As can be predicted from the population peristimulus time histograms, both peak firing rate and excitatory area under the curve elicited by trapezoidal stimuli were significantly greater in sham than TBI animals at most ramp velocities in all laminae, except Layer 5. A suppression of peak firing rate in TBI cases was found in Layer 2 at the two largest ramp velocities, and in Upper Layer 3, Deep Layer 3, and Layer 4 at all ramp velocities except the slowest (Two-way ANOVA, *p*<0.05). A reduction in excitatory area under the curve in TBI animals was observed in Layer 2 at the two highest ramp velocities, in Upper and Deep Layer 3 at the four highest ramp velocities and in Layer 4 at all tested velocities. Overall, there was a depth-dependent suppression of neuronal firing, with the greatest dampening of responses occurring towards the cortical surface.

**(b) TBI effects on response strength to complex whisker motions:** For the rough surface discrimination stimulus, recordings were obtained from a total of 66 responsive multi-units from TBI animals and 47 responsive multi-units from sham animals. There was no significant difference between the two groups in numbers of responsive clusters in each layer (χ^2^ = 3.09, df = 4, *p*>0.05). For the object contact stimulus, recordings were obtained from a total of 66 responsive multi-unit clusters in TBI animals and 50 responsive multi-unit clusters in sham animals; again there was no significant difference between the two groups in numbers of responsive clusters in each layer (χ^2^ = 2.90, df = 4, *p*>0.05).

**Figure 4:**
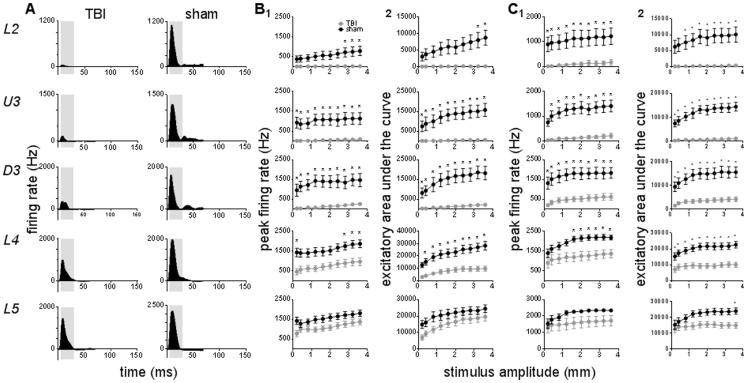
TBI effects on barrel cortical responses evoked by complex stimuli applied to the PW. Population Grand peristimulus time histograms are shown in (**A)** in response to the rough surface discrimination motion stimulus with the highest amplitude (3.6 mm) in a specific lamina (lamina indicated to left of panel) in TBI and sham animals. Laminar designations: L2 = Layer 2; U3 = Upper Layer 3; D3 = Deep Layer 3; L4 = Layer 4; L5 = Layer 5. Each Grand peristimulus time histogram was generated by averaging responses across all responsive clusters in that lamina. The grey shaded box represents the analysis window used to extract various response onset metrics. (**B**) depicts the response metrics derived from the analysis window for the rough surface discrimination motion stimulus (for which Grand peristimulus time histograms are shown in A) and (**C**) depicts the response metrics derived from the analysis window for the object contact motion stimulus (see text). In (**B**, **C**), column 1 is the Peak firing rate and column 2 is the excitatory area under the curve extracted from the onset response to simple trapezoidal stimuli. Data represents averages from all responsive clusters (±SEM) at all tested ramp velocities, separated by cortical lamina. Each row of data comes from the same lamina as designated by the labels on the left. In each panel data from clusters in TBI animals is shown as the grey circles and that in Sham surgery animals as the black circles. (*) *p*<0.05.

As with the simple trapezoidal deflections, a marked suppression of responses was also apparent following application of complex stimuli in TBI cases. For brevity, we present only the Grand peristimulus time histograms for the rough surface discrimination stimulus obtained at the highest stimulus amplitude (3.6 mm; Fig. 4A); very comparable effects were also observed for the object contact stimulus and so it is not illustrated.

The pattern of activity to the rough surface discrimination stimulus consisted, in sham surgery animals, of a single large peak at stimulus onset in all layers, followed by low level tonic excitation in all layers from Layer 2 to Layer 4, and offset inhibition in Layer 5. As with the case of the trapezoid stimuli, in TBI cases the pattern of responses was similar to that in the sham surgery animals, except that there was a clear reduction in response amplitude in all layers and no observable offset inhibition (in Layer 5).

To characterise the response metrics to the onset of the stimuli, an analysis window of 5–50 ms from stimulus onset was applied for the object contact stimulus and a shorter analysis window of 5–30 ms from stimulus onset (to encompass the entire stimulus duration) for the rough surface discrimination stimulus was used (grey shaded box, Fig. 4A). In response to the rough surface discrimination motion stimulus, a reduced peak firing rate was observed in TBI animals in Layer 2, Upper Layer 3 and Deep Layer 3 at all whisker motion amplitudes (except for the lowest amplitude in Upper Layer 3) and in Layer 4 at the 6 highest whisker motion amplitudes (Fig. 4C; Two-way ANOVA, *p*<0.05). The excitatory area under the curve was narrower in TBI animals at all stimulus amplitudes in Upper Layer 3, Deep Layer 3 and Layer 4, and to all but the lowest two amplitudes in Layer 2 (Two-way ANOVA, *p*<0.05). No change in peak firing rate or excitatory area under the curve was observed in Layer 5 between TBI and sham surgery animals following this type of stimuli.

Similarly, for the object contact whisker motion stimulus, peak firing rate was significantly dampened in TBI animals at all stimulus amplitudes in Upper Layer 3 and Deep Layer 3, with a significant suppression in Layer 2 and Layer 4 only at the 3 largest whisker motion amplitudes (Fig. 4B; Two-way ANOVA, *p*<0.05). Excitatory area under the curve was also reduced in TBI animals, at all but the lowest stimulus amplitude in Upper and Deep Layer 3, Layer 4, and following the 2 largest stimulus amplitudes in Layer 2 (Two-way ANOVA, *p*<0.05). There was no difference in response firing parameters in Layer 5 between TBI and sham animals.

In summary, across all three stimuli, we observed a suppression of firing rate and a narrowing of response dispersion in TBI animals in a depth-dependent manner (Table 1).

### Absence of Changes in Temporal Metrics Following TBI

In contrast to these TBI-induced changes in firing rate, we found that there were no systematic changes in any temporal measures of neuronal responses to any of the three stimuli. The two temporal measures we calculated were the latency from the stimulus onset to the peak firing rate (latency to peak firing rate; Supplementary Figure S1) and the temporal dispersion of the onset peak (half-peak width; Supplementary Figure S2), measured as the duration in the analysis window over which firing rates were ≥50% of the peak firing rate. There were no differences in latency to peak firing rate between TBI and sham surgery animals at any ramp velocity for the trapezoidal stimuli, or any stimulus amplitude for the object contact and rough surface discrimination stimuli. A reduction in half-peak width was detected in TBI animals in response to all three stimuli, but only in layers where responses were heavily dampened by injury, and predominantly in response to smaller stimulus ramp velocity and amplitude that consequently evoked very small response events (Two-way ANOVA, *p*<0.05). This detected change was presumably a result of the small firing rate in these layers (see Figs. 3 and 4), and not a direct result of changes in thalamocortical timing or connectivity.

### Enhanced Spontaneous Firing Rate in Cortical Layer 4 and 5 Following TBI

**Figure 5:**
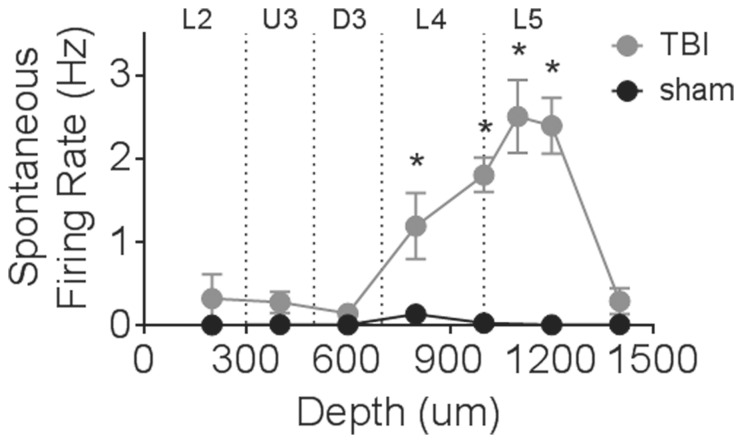
Elevated spontaneous firing rate following TBI. Spontaneous firing rate was measured in the 200 ms window prior to stimulus onset, and was extracted from single-unit data for TBI (n = 170 single cells) and sham (n = 115 single cells) animals. The figure plots the mean spontaneous firing rate in clusters grouped according to depth from the cortical surface. Depth-boundaries of the laminae are shown. Depth-dependent changes in spontaneous output were observed in TBI animals only in Layer 4 and Layer 5a, with spontaneous firing rate returning to baseline sham levels in Layer 5b (at 1400 µm from the cortical surface).

Major neuronal reorganisation as occurs following various types of injury is often associated with increased levels of spontaneous activity [46], [47], and is also reported to occur in the immediate period following TBI [48]–[50]. In order to assess whether changes in spontaneous firing rate were present 24 hours post-TBI we sorted single cell data from the multi-unit clusters using standard Spike 2 template matching algorithms, applied to each stimulus amplitude. Spontaneous firing rate (recorded in the 200 ms window prior to stimulus onset) was extracted from this single-unit data for both TBI and sham animals. Interestingly, elevated spontaneous output was measured in Layer 4 and Layer 5A only (Figure 5; TBI_L4_ = 1.49±0.30 Hz; sham_L4_ = 0.07±0.01 Hz; TBI_L5_ = 2.44±0.38 Hz, sham_L5_ = 0.007±0.004 Hz; Two-way ANOVA, *p*<0.05), with spontaneous firing rate returning to sham levels at a depth of 1400 µm. This is in direct contrast to our previous data obtained at 8–10 weeks post-injury in which no difference in spontaneous firing rate was observed in TBI animals [18], and provides useful insight into the mechanisms underlying circuit remodelling in the early stages following injury.

### Effects in an Uninjured WDIA-treated Animal: Behaviour and Neuronal Responses

**Figure 6:**
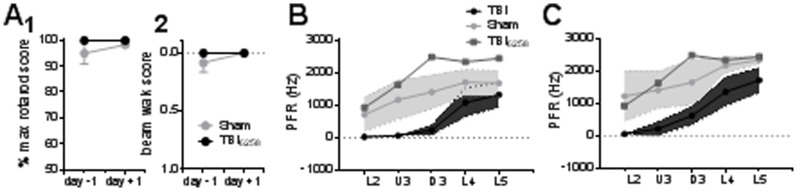
Behavioural deficit as a predictor of neuronal function. Changes in behaviour pre- (day −1) and post- (day +1) surgery for TBI animal 6258, which exhibited no behavioural deficit as determined by rotarod (**1**) and beam walk (**2**) tasks. (**B, C**) Peak firing rate at the highest velocity trapezoidal stimulus (**B**) or at the highest amplitude rough-surface-discrimination motion stimulus (**C**) in each lamina for the 7 other WDIA-treated animals that showed behaviour deficits (labelled TBI here), the sham surgery control animals, and the one WDIA-treated animal that showed no behaviour deficit (TBI animal 6258). For the groups (i.e., the 7 TBI animals and the 6 Sham control animals) the shaded regions represent the 95% confidence interval about the mean (dots). Data from animal 6258 is very closely aligned with that of the sham animals, and is much higher than observed in the other WDIA-treated animals showing behavioural deficits. Behavioural deficit may therefore be a useful predictor of neuronal output. L2 = Layer 2; U3 = Upper Layer 3; D3 = Deep Layer 3; L4 = Layer 4; L5 = Layer 5.

The above data on TBI were derived from 7 of 8 WDIA-treated animals. We excluded from that analysis one WDIA-treated animal that exhibited no behavioural deficit at 24 hours post-treatment. Although this animal received the standard WDIA treatment, and although it was within the same age and weight range as the other animals, at 24 hours post-treatment, it showed absolutely no deficits in either rotarod test or the beam walk test (Fig. 6A). After the behaviour tests, electrophysiological data was also obtained from this animal at 24 hours post-treatment, as in the other 7 TBI animals and the 6 sham surgery animals.

Congruent with its normal behaviours, in this animal responses from barrel cortex neurons evoked by simple and complex stimuli were similar to the responses seen in sham animals (Fig. 6B, C). We calculated 95% confidence intervals for the two surgical treatments, and found that data obtained from this atypical TBI animal fell within the peak firing rate range predicted by the sham data, and remained well above that predicted by the remaining TBI data.

While we cannot identify why this animal showed no behaviour deficits or neuronal response changes to the standard injury method that caused behaviour deficits and large neuronal response changes in the other 7 animals, the absence of changes in barrel cortex neuronal responses in this animal is consistent with the total absence of behaviour deficits in the animal. These results act as a counterpoint to the data from the other 7 TBI animals in linking neuronal response changes and behaviour deficit in TBI.

### Comparison between Short- and Long-term Deficits in Sensory Processing Following TBI

The results presented here are in stark contrast to those previously described by us at 8–10 weeks post-TBI using the same method of inducing TBI [18]. We used the same suite of trapezoidal stimuli and the same two complex stimuli as in that study (as well as another two complex stimuli) and data were collected and analysed in the same way, allowing for direct comparison of effects in the two studies which differed only in the time when recordings were obtained post-treatment. Here we compare only the population peak firing rate data in the two studies to the trapezoidal stimuli and the two complex stimuli common to the two studies; these data have not been reported previously.

**Figure 7:**
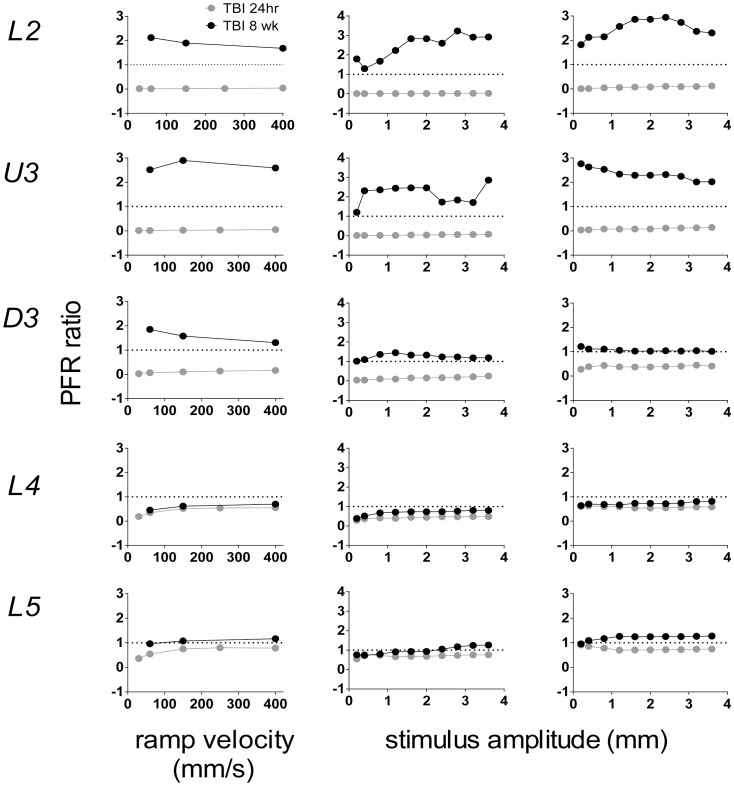
Comparison of the TBI-induced short- and long-term neuronal changes in peak firing rate to all three whisker stimuli used in this study. Each panel compares the TBI-induced changes in peak firing rate, relative to the Sham surgery controls, at 24 hours post-WDIA treatment and 8–10 weeks post-WDIA treatment. Ratio between mean peak firing rate in TBI animals to the mean peak firing rate in sham animals is shown. A ratio of 1 indicates that firing rates in the two groups were the same. The pattern of whisker stimulation is shown above the uppermost panel for the trapezoid (left-hand panels), object contact (middle panels) and rough surface discrimination (right-hand panels) whisker motions. At 24 hrs post-TBI there is a depth-dependent suppression of neuronal responses in TBI animals (n = 7) relative to their sham surgery counterparts (n = 6), however at 8 weeks after TBI there is hyperexcitation to the same stimuli in TBI animals (n = 16) in the upper layers, when compared with sham surgery controls (n = 14). Data at 8–10 weeks post-TBI taken from Alwis D *et al*., (2012).

For each stimulus type, to allow for easy comparison of effects at 24 hours against those at 8 weeks, we calculated the ratio of the mean peak firing rate in TBI animals at that time point to the mean peak firing rate in the sham surgery control animals for the same time point. This allows for each time point to be referenced to its own Sham surgery group. As shown in Figure 7, the effects of TBI relative to the Sham surgery animals are markedly different at the two time points, particularly in the upper layers where *hypoexcitable* responses at 24 hours post-injury are in stark contrast to *hyperexcitable* responses by 8–10 weeks post-injury. Collectively these data suggest that there must be a complex rearrangement of circuitry that occurs between 24 hours and 8–10 weeks post-injury, with an initial depression of responses immediately following impact, followed by a recovery period in which responses surpass their initial excitability level, and a heightened level of excitability is established in the circuit.

### Immunohistochemical Confirmation of Diffuse Brain Injury

**Figure 8:**
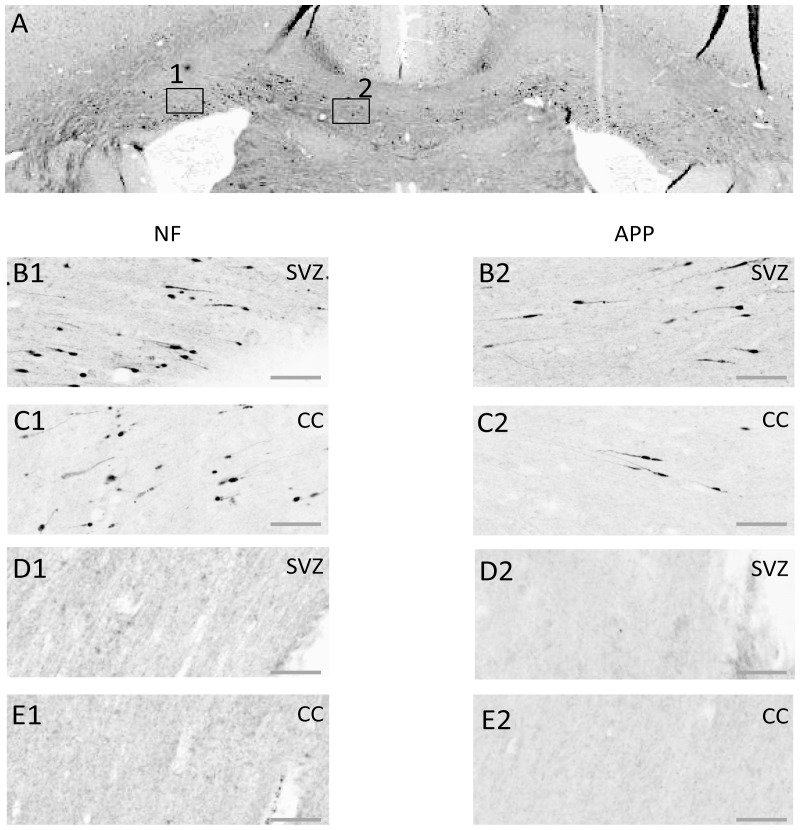
Axonal injury as indicated by immunohistochemical staining for neurofilament heavy-chain (“NF-H”) OR β-amyloid precursor protein (APP). Exemplar coronal section (x6 magnification) 1.3 mm caudal of bregma analysed for staining for NF-H and for β-APP is shown in (**A**). The boxes indicate the sub-ventricular zone (Box 1; SVZ) or the corpus callosum (Box 2; CC) shown in greater detail (x20 magnification) in the next four rows of panels. (**B, C**) Staining in a TBI animal 24****hrs post-TBI for NF-H (B1, C1) or β-APP (B2, C2) in the SVZ (row B) or the CC (row C). (**D, E**) As for (**B, C**) for equivalent brain regions, from a Sham control animal. Scale bar indicates 50 µm.

Diffuse axonal injury can be indexed by axonal swelling and bulbs, primarily in white matter. We used immunohistochemistry for neurofilament heavy-chain (NF-H; 200 kDa) and for β-amyloid precursor protein (β-APP) to identify axonal injury after electrophysiological recordings were completed. Prototypical results are shown in Figure 8, which presents results from one TBI and one sham animal, both stained for NF-H (left column of photos, B1 and C1, labelled “NF”) or for β-APP (left column of photos, B2 and C2, labelled “APP”) in the sub-ventricular zone (label “SVZ”, box 1 in Fig. 8A) and in the corpus callosum (label “CC”, box 2 in Fig. 8A). These results agreed with our previous observations based on the same injury model [35] that diffuse axonal damage could be identified through staining of NF-H and β-APP in the corpus callosum, sub-ventricular zone, external capsule and cingulum. Figure 8 also presents equivalent histological data for a Sham surgery animal (D1, D2, E1 and E2) to show the absence of staining for NF-H (D1, E1) or for β-APP (D2, E2) in the same brain regions. A further link between the immunohistochemistry and cortical and behaviour outcomes was found from histological examination of the brain taken from the animal that exhibited no behavioural or electrophysiological deficit following injury (discussed above) which revealed a lack of positive staining for NF-H and β-APP in the corpus callosum, and only marginal staining for both markers in the sub-ventricular zone (see Supplementary Figure S3 and compare to Fig. 8 D1, D2, E1, E2). This provides further confirmation for the link between extent of injury and neuronal function. In the present study, in all TBI animals (bar the one anomalous animal) and in none of the sham surgery animals there was consistent axonal swelling and bulbs in all four of these brain areas.

## Discussion

In this study we recorded responses from the rat whisker-barrel cortex system to gain insight into deficits in sensory cortical neuronal processing associated with TBI. Twenty four hours post-injury, we found hypo-excitation in neuronal responses to both simple and complex stimuli in upper and input layers, concurrent with elevation in spontaneous activity in Layers 4 and 5a. These findings are in stark contrast to the effects we have previously described at 8 weeks post-TBI, in which hyper-excitability was noted but only in supra-granular layers and markedly to complex rather than simple stimuli [18].

There was no difference in the number of clusters in each lamina in TBI animals compared to the Sham surgery animals. Thus the change in population response strength in a lamina was not due to a lower number of responsive elements but due to a decrease in the responsiveness of the recorded elements. Nor were there any systematic major differences in temporal measures of responsiveness to any stimuli, suggesting that the change in cortical responsiveness was a local phenomenon, not one relayed from sub-cortical sites. Consistent with the conclusion of local phenomena are the facts that there was a clear depth-dependency to the change in responsiveness and there was no change in Layer 5 response strength to any of the three whisker motion stimuli we used.

### Stress Wave Phenomena may Cause Ionic Imbalances that Result in Neuronal Suppression in Concussive Impact/acceleration Brain Injury

In the present study, 24 hours post-TBI there was a suppression of responses in rodent barrel cortex in a depth-dependent manner, with greatest effects in the most superficial layers and decreasing systematically with cortical depth. In contrast to our previous observations at 8 weeks post-TBI [18] this effect was independent of sensory stimulus pattern (complexity) Therefore the extent of neuronal responsiveness is not determined by the properties of the stimulus delivered, but the suppression is indicative instead of an inherent dysfunction within the circuit that occurs independent of input type. A suppression of cortical responses immediately after brain injury has been reported in Layer 4 cortical neurons (the only layer studied there) between 5 to 20 minutes after injury [16]. In a similar vein, c-Fos staining shows an initial reduction in cortical activity post-TBI [51] These “global” depth-dependent suppressive effects are consistent with the strain (stress) wave known to propagate through the skull and underlying brain tissue on head impact [17], [30]–[34].It has been reported that by 3 days after brain injury, evoked potentials from somatosensory cortex have significantly longer latencies and reduced field potential slopes [52], which is in line with decreased metabolic activation that occurs as early as 4 hours and up to 24 hours after injury [53]. Beyond these studies, little is known of the electrophysiology of cortical responses in the near-term post TBI, and our study is the first to examine details across the columnar network of sensory cortex. In the cortical compression TBI study by Ding *et al*. [16], the immediate-post-injury suppression was attributed to disruptions of ionic homeostasis and to spreading depression. It appears likely then that the suppression in the neuronal population responses to all stimulus types we observed may also result from immediate changes in ionic balances in neurons and glial cells, but here caused by a stress wave transmitted through the brain.

It is important to note that the depth-dependent neuronal suppression we observe may also result from different cellular vulnerability to injury between the laminae, depending on the dominant excitatory cell type. Indeed, different brain regions are known to exhibit different susceptibility to injury, and this is further complicated by the injury type experienced [54], [55]. Studies on the hypotensive brain have demonstrated that Layer 3 neurons in cortex are most susceptible to ischemic insult [56], but caution must be exercised when drawing parallels between these data and our own. We have shown that the hypoactivity appeared to be nearly complete in Layer 2 and then effects decreased in a systematic depth-dependent manner. This suggests that the primary determinant is the propagation of the stress wave through the brain tissue, and this is unlikely to be selective for cell type, at least at this early post-injury stage. This is consistent with data from the cortical compression TBI study by Ding et al. [16] where there is also a uniform suppression of responses immediately post-TBI (the order of minutes in that study). If the effect is due to cortical spreading depression, as suggested in that study, it is difficult to see that this effect would be selective as to which neurons it would affect.

One other line of evidence for the non-selectivity of the effects responsible for the hypoactivity we observe is seen in the fact that depth-dependent neuronal suppression was seen for all stimulus types tested here. This is much more consistent with dysfunction of all elements of the cortical circuit, i.e., to both excitatory and inhibitory neurons, in contrast to the long-term effects of hyper-excitation [18] which is more likely to be due to selective neuronal changes resulting in changes in the excitation – inhibition balance. In summary we conclude it possible that differences in cortical cytoarchitecture (perhaps in combination with stress wave phenomena) may also be important in establishing the lamina-specific changes we report here. However, neither our data nor that of Ding et al. [16] can currently resolve this issue and, for now, we can only state unequivocally that the resultant “global” neuronal dysfunction we observed was not rescued by alternate stimuli properties, like velocity or vibratory changes, and instead is comparable to a cortical silencing that remains despite the afferent input.

### Development of Hyperexcitability from an Initially Hypoexcitable State

Our current studies (present study and [18]) suggest that following the stress wave-driven suppression of cortical responsiveness, there is neuronal recovery in the following weeks, but this results in an overshoot to produce supragranular hyperexcitability at 8–10 weeks post injury. Other examples of an initial hypo-excitable state to a hyper-excitable state have been described above in other models of injury. Ding *et al*., [16] found that after the initial suppression of cortical responses (studied only in Layer 4 cortical neurons in their case) at between 5 to 20 minutes after injury, there was a period of *hyper*-activity at approximately 2 hours post-injury. Similarly, the initial suppression of cFos staining post-TBI is followed by a sustained period of hyperactivity in cortex, hippocampus and thalamus [51].

In combination, these data suggest that shifts in the excitation/inhibition balance in the cortex are likely a common phenomenon following various types of injury [57]. As noted by Ding *et al*., [16], the general consensus of studies attempting to ascribe mechanisms to the hyperexcitability focuses on changes in GABA mechanisms, especially tonic GABA signalling through GABA_A_ [57].

The development of a hyperactive state following stroke is largely attributed to disinhibition [58], [59], which is characterised by reduced GABAergic activity [60], [61], occurs in the first 24 hours following the ischemic attack [60], [62], and is associated with substantial neuronal plasticity [60]. In head trauma, a number of short-term complex changes act to ‘prime’ the system for more established, permanent changes [63]. The initial traumatic impact likely leads to a spreading depression, characterised by a depolarisation of neurons and glia lasting 1–2 mins [63]–[66]. Spreading depression moves in a wave-like fashion across grey matter, and is dependent on NMDARs for its propagation [67], [68]. Mechanical deformation of the brain tissue also occurs upon impact, leading to disruption of inward and outward neuronal membrane currents and elevated extracellular K^+^, which induces a self-propagating depolarisation throughout the linked cortical networks upon cessation of the wave [64]. More permanent mechanisms are required to sustain long-term hyperexcitability, and several distinct mechanisms are thought to be critical in establishing this. Changes in intrinsic neuronal properties have been documented over a period of weeks following injury, and include alterations to A-type K^+^ currents [10] and impaired glial K^+^ homeostasis leading to increases in extracellular K^+^ concentration [69]. Perturbations to inhibitory neurotransmission have also been reported following TBI [70], with a reduction in spontaneous and miniature IPSC frequency at 8–13 weeks after injury [71]. Similar to the aforementioned stroke studies, a progressive loss of GABA neurons has also been reported following TBI, lasting up to 6 months post-injury [72]. Others have documented the onset of aberrant axon sprouting following injury, leading to an increase in the number of excitatory connections between neurons [73]–[75]. It is likely that combinatorial effects of increased excitability (as a result of changes in intrinsic neuronal properties and extracellular milieu), disinhibition and increased excitatory connectivity via aberrant axonal sprouting all contribute to the development of hyperactivity within the cortex following brain injury.

### Increased Spontaneous Discharge in Layers 4 and 5

At 24 hours post-trauma, a significant increase in the rate of spontaneous output was noted in Layer 4 and part of Layer 5. Such increases in non-evoked release are commonly reported in various types of TBI models [48]–[50], and have previously been linked to post-TBI hyperexcitability at 2–6 weeks post-injury [76]. The increase in spontaneous firing rate we report here is of particular significance, for two major reasons. Firstly, increased spontaneous discharge following TBI is usually only described in the hippocampus (where it is thought to play a role in establishing the hippocampal formation as a focus for posttraumatic seizures [49]) and up until now had not been described in the barrel cortex. Secondly, the presence of increased spontaneous firing rate in Layer 4 neurons, despite the dominant suppression of neuronal responses that is observed in the population at this site 24 hours post-injury provides intriguing insight into the underlying cellular changes that are likely occurring. There must be unique subsets of cells that exhibit enhanced release, with the majority being depressed and dominating the population response.

Given the complex intra-lamina connections that exist in a functional barrel column [77]–[79], it is difficult to postulate which specific cell types are responsible for the enhanced output. Nevertheless it is interesting to speculate about which aspects of the network may be involved. One limitation of our recording method is that single-unit metrics (to perform spontaneous rate analysis) can only be extracted from large-cell types. Therefore it follows that it must be inputs to these large cells that exhibit an enhanced spontaneous output. In Layer 4 there are three distinct cell types: spiny stellate cells, star pyramids and Layer 4 pyramidal neurons [80]–[82]. Each cell type receives a complex pattern of inputs from a number of different sources, with inputs from VPM neurons in the thalamus dominating the circuit [83], [84]. Layer 5 contains at least two main excitatory cells types, with a further division into sublaminae on the basis of location of these cells. Layer 5A contains slender tufted pyramidal neurons [84]–[89] which receive unique thalamic input from the medial posterior (POm) nucleus as part of the paraleminscal sensory pathway [90] in addition to its major Layer 2/3 drive. Layer 5B contains thick-tufted pyramidal cells [91] which receive thalamic input from the ventral posteromedial (VPM) nucleus to form part of the lemniscal sensory pathway [92], [93]. Of particular relevance for our findings is a large input that exists from Layer 4 spiny stellate cells to Layer 5A pyramids [80], [94]. We postulate that the enhanced spontaneous drive we record in Layers 4 and 5A originates from the VPM inputs to Layer 4 spiny stellate cells, and is then conveyed via these cells into Layer 5A. There is no such connection between Layer 4 spiny stellates and Layer 5B pyramids [94]. This would allow for a small sub-network of cells within a single columnar unit to dictate a heightened level of activity to the various output structures from Layer 5, and may help establish the hyperexcitability to various stimuli that has been recorded following TBI by us and others at later time points post-injury [10], [16], [18], [48], [95]. Therefore the enhanced spontaneous release rate that is evident immediately post-injury may act to ‘reset’ the upper layer network into a hyperexcitable state in the long-term.

### Clinical Significance

The direct clinical relevance of our data is seen in the type of injury model and the parameters we used to create TBI with that model. The closed head impact WDIA injury method [17] used to create diffuse TBI here allows replication of the factors and effects that impact on the head and brain during a blow to the human head and has strong face and construct validity with human motor vehicle and sporting field accidents. We therefore postulate that the complex and dynamic changes in processing in sensory cortex that we describe here in the short-term and described previously for the long-term [18] must also occur in humans suffering blows to the head.

Additionally, we suggest that changes in cortical sensory processing must underpin at least some human behavioural deficits post-TBI. Deficiencies in auditory, visual and tactile processing have been reported following brain injury [96], [97], and survivors of brain injury display a heightened sensitivity to sensory stimuli [27]–[29]. In an experimental model of TBI, a similar behavioural hypersensitivity has been documented to sensory whisker stimulation in rodents at 4–8 weeks post-injury [98], [99]. As already noted, neuronal hyper-sensitivity post-TBI has been demonstrated in sensory cortex by Ding *et al*. [16] following cortical compression injury: an initial response suppression in barrel cortex Layer 4 neurons at 5–20 mins post-injury was followed by a hyper-excitation that gradually developed over the next 2 hours. However, no data was presented beyond this 2 hour period and it is therefore not known how long this hyper-excitation persisted and consequently its relevance to the sensory hyper-sensitivity seen in persistent long-term TBI.

We postulate that avenues of treatment that halt the progression of the changes we describe will prove beneficial in human TBI. Therapeutic areas of particular interest will target the secondary effects that follow the spreading depression wave, with the aim of preventing the excitation/inhibition imbalance that occurs following TBI. Drugs that reduce changes in the extracellular environment may prove especially beneficial, and a particularly novel avenue may be to investigate a means of normalising K^+^ levels by restoring activity of the inward rectifier K^+^ channels in glia. Restoration of this inward current may encourage maintenance of normal neuronal excitability. Similarly, drugs that act to restore normal inhibitory function within the circuitry may prove beneficial. Studies using a stroke-model of injury report beneficial neuroprotective effects of the GABA_A_ receptor agonist muscimol and the GABA_B_ receptor agonist baclofen, particularly when used in combination [100], [101]. Further histological studies have supported the neuroprotective role of these agonists [102], and while they appear to offer some regain of motor function when delivered intrathecally post-TBI [103]–[105], their role in restoring cognitive function has yet to be assessed. Finally, attempts have been made to inhibit the axonal sprouting that occurs following injury, in the hope that this will prevent the onset of hyperexcitability. In one recent study, blockage of collapsin response mediator protein 2 (CRMP2; an axonal guidance protein) prevented enhanced synaptic connectivity following injury [106]. Part of the challenge arises from the large number of target sites within the circuitry that could be manipulated to restore the network imbalance. Nevertheless, it remains clear that therapies that restore or lessen this imbalance will likely improve patient outcome in the immediate period following TBI.

## Supporting Information

Figure S1 **Effects of TBI on Latency to peak firing rate.** This temporal measure is presented for the responses to simple trapezoidal **(A)**, complex object contact **(B)** and rough surface discrimination **(C)** stimuli. Metrics were extracted from the onset response. All values represent averages (±SEM) from all responsive clusters in the various lamina for TBI (grey circles) and sham surgery animals (black circles). L2 = Layer 2; U3 = Upper Layer 3; D3 = Deep Layer 3; L4 = Layer 4; L5 = Layer 5.
(TIF)

Figure S2 **Effects of TBI on half-peak width.** This temporal measure, of the width of the onset peak at half the peak firing rate, is presented for the responses to simple trapezoidal **(A)**, complex object contact **(B)** and rough surface discrimination **(C)** stimuli. Metrics were extracted from the onset response. All values represent averages (±SEM) from all responsive clusters in the various lamina for TBI (grey circles) and sham surgery animals (black circles). L2 = Layer 2; U3 = Upper Layer 3; D3 = Deep Layer 3; L4 = Layer 4; L5 = Layer 5. (*) *p*<0.05.
(TIF)

Figure S3 **Lack of axonal injury in animal subjected to TBI treatment, but with no apparent electrophysiological or behavioural deficit.** Axonal injury was assessed by immunohistochemical staining for neurofilament heavy-chain (“NF”) OR β-amyloid precursor protein (APP). **(A)** Staining in the SVZ and **(B)** CC with NF-H (**A1, B1)** and β- APP **(A2, B2)** at 24****hrs post-TBI in an animal that exhibited no behavioural or electrophysiological deficit. Complementary to this, no noticeable staining was noted for either NF-H or β- APP. See Figure 9A for example coronal section from which these regions were extracted. Scale bar indicates 50 µm.
(TIF)

Table S1 **Results of Two-way repeated measures ANOVA statistical analysis of peak firing rate, excitatory area under the curve, latency to peak firing rate and half-peak width in clusters responsive to the trapezoidal stimulus from 5–50 ms from stimulus onset (related to** **Figures 3** **and** **5A** **).** The Table lists F statistics and degrees of freedom for both significant and non-significant factors for main and interaction terms.
(DOCX)

Table S2 **Results of Two-way repeated measures ANOVA statistical analysis of peak firing rate, excitatory area under the curve, latency to peak firing rate and half-peak width in clusters responsive to the object contact stimulus from 5–50 ms from stimulus onset (related to** **Figures 4B** **and** **5B** **).** The Table lists F statistics and degrees of freedom for both significant and non-significant factors for main and interaction terms.
(DOCX)

Table S3 **Results of Two-way repeated measures ANOVA statistical analysis of peak firing rate, excitatory area under the curve, latency to peak firing rate and half-peak width in clusters responsive to the rough surface discrimination whisker motion stimulus from 5–30 ms from stimulus onset (related to** **Figures 4C** **and** **5C** **).** The Table lists F statistics and degrees of freedom for both significant and non-significant factors for main and interaction terms.
(DOCX)
