# Supplementary figures and images for: Cortical Hypoexcitation Defines Neuronal Responses in the Immediate Aftermath of Traumatic Brain Injury
Source: PLoS One. 2013 May 7;8(5):e63454. doi: 10.1371/journal.pone.0063454 (PMC3646737; doi:10.1371/journal.pone.0063454)

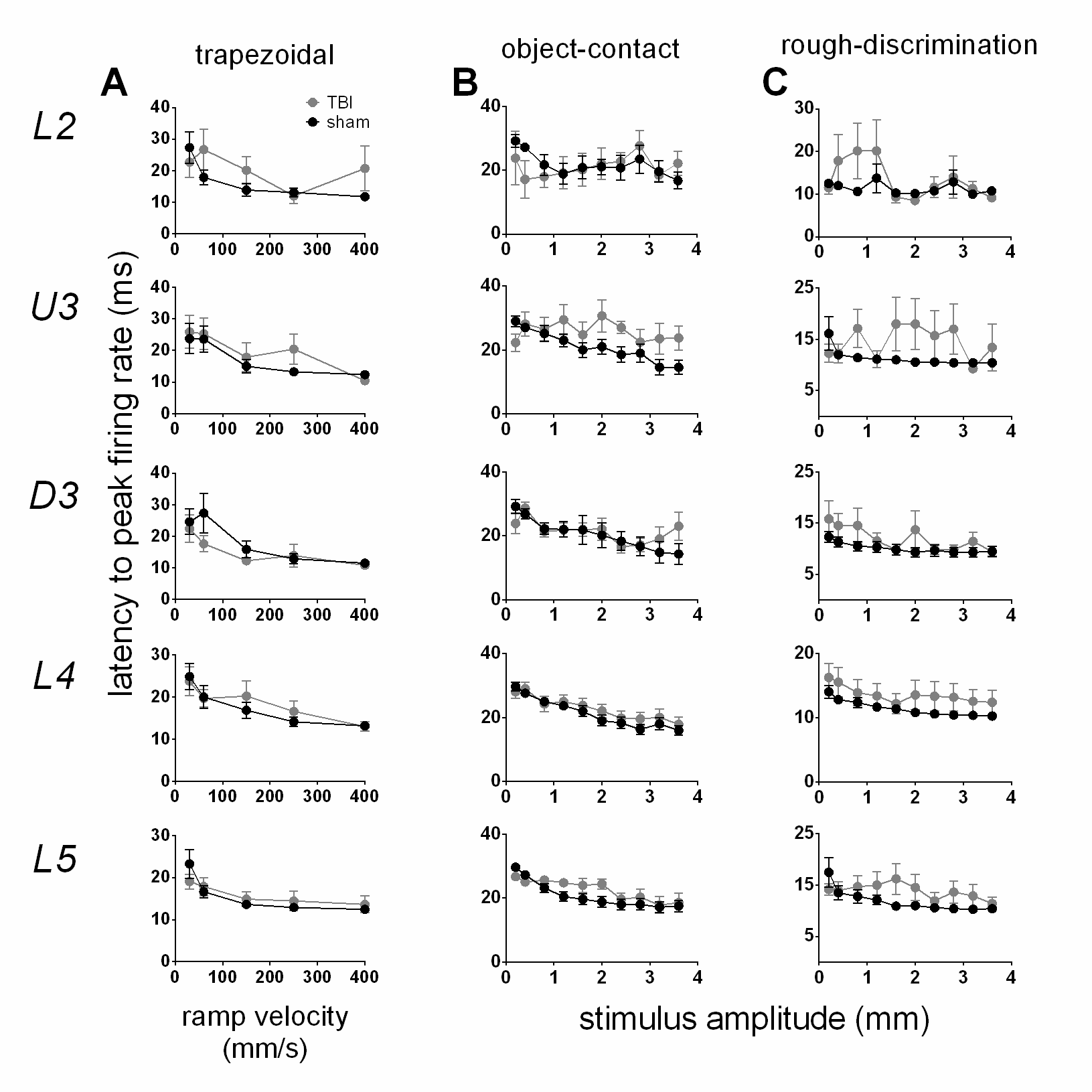

Supplement: Figure S1 — Effects of TBI on Latency to peak firing rate. This temporal measure is presented for the responses to simple trapezoidal (A), complex object contact (B) and rough surface discrimination (C) stimuli. Metrics were extracted from the onset response. All values represent averages (±SEM) from all responsive clusters in the various lamina for TBI (grey circles) and sham surgery animals (black circles). L2 = Layer 2; U3 = Upper Layer 3; D3 = Deep Layer 3; L4 = Layer 4; L5 = Layer 5. (TIF) [file pone.0063454.s001.tif]

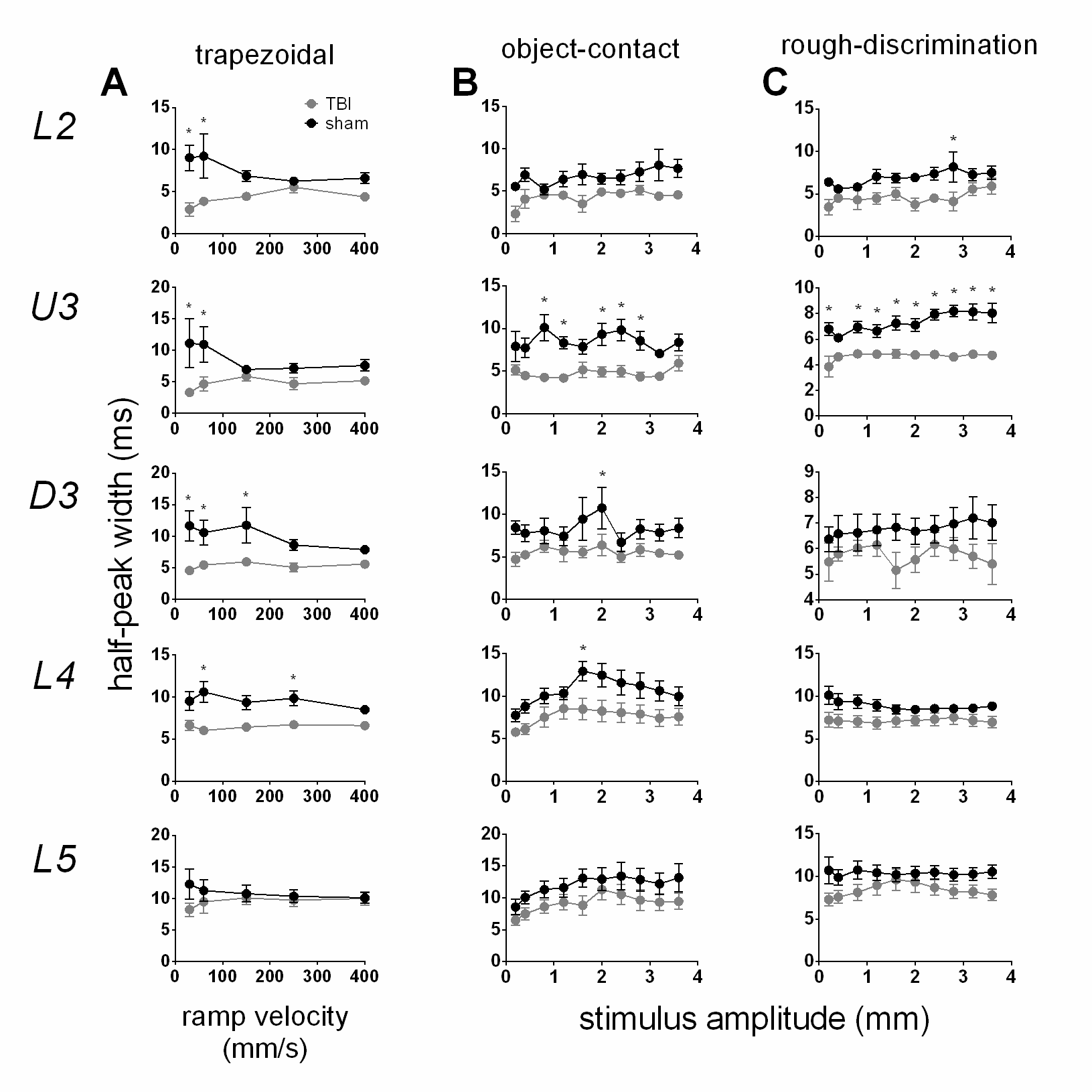

Supplement: Figure S2 — Effects of TBI on half-peak width. This temporal measure, of the width of the onset peak at half the peak firing rate, is presented for the responses to simple trapezoidal (A), complex object contact (B) and rough surface discrimination (C) stimuli. Metrics were extracted from the onset response. All values represent averages (±SEM) from all responsive clusters in the various lamina for TBI (grey circles) and sham surgery animals (black circles). L2 = Layer 2; U3 = Upper Layer 3; D3 = Deep Layer 3; L4 = Layer 4; L5 = Layer 5. (*) p<0.05. (TIF) [file pone.0063454.s002.tif]

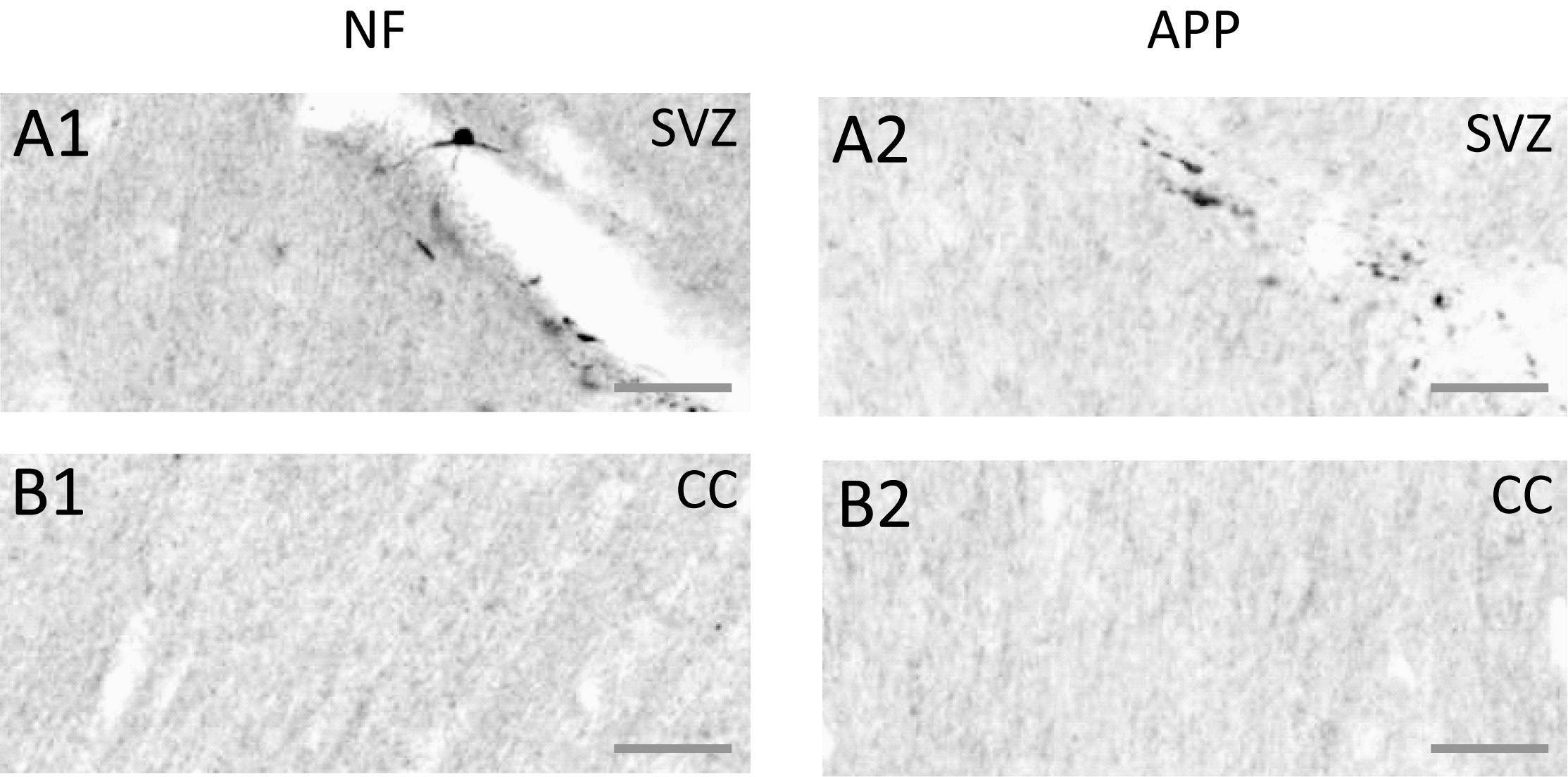

Supplement: Figure S3 — Lack of axonal injury in animal subjected to TBI treatment, but with no apparent electrophysiological or behavioural deficit. Axonal injury was assessed by immunohistochemical staining for neurofilament heavy-chain (“NF”) OR β-amyloid precursor protein (APP). (A) Staining in the SVZ and (B) CC with NF-H (A1, B1) and β- APP (A2, B2) at 24hrs post-TBI in an animal that exhibited no behavioural or electrophysiological deficit. Complementary to this, no noticeable staining was noted for either NF-H or β- APP. See Figure 9A for example coronal section from which these regions were extracted. Scale bar indicates 50 µm. (TIF) [file pone.0063454.s003.tif]
