# Supplementary material for: Cortical Hypoexcitation Defines Neuronal Responses in the Immediate Aftermath of Traumatic Brain Injury
Source: PLoS One. 2013 May 7;8(5):e63454. doi: 10.1371/journal.pone.0063454 (PMC3646737; doi:10.1371/journal.pone.0063454)
Supplement: Table S1 — Results of Two-way repeated measures ANOVA statistical analysis of peak firing rate, excitatory area under the curve, latency to peak firing rate and half-peak width in clusters responsive to the trapezoidal stimulus from 5–50 ms from stimulus onset (related to Figures 3 and 5A ). The Table lists F statistics and degrees of freedom for both significant and non-significant factors for main and interaction terms. (DOCX) [file pone.0063454.s004.docx]

**Supplementary Data**

**Table 1. Results of Two-way repeated measures ANOVA statistical analysis of firing rate (PFR), excitatory area under the curve (EAUC), latency to PFR and half-peak width (HPW) in clusters responsive to the trapezoidal stimulus from 5-50ms from stimulus onset (related to Figures 3 and 5A).** The Table lists F statistics and degrees of freedom for both significant and non-significant factors for main and interaction terms.

| Response metric: Peak excitatory firing rate (PFR) in the onset response analysis window from 5-50 ms from stimulus onset**.** | | |
| --- | --- | --- |
| **Layer** | **Main terms** | **Interaction terms** |
| L2 | Group *F*_1,14_ = 5.12, *p* = 0.04  Amplitude *F*_4,56_ = 10.13, *p* < 0.0001 | Amplitude x Group *F*_4,56_ = 8.48, *p* < 0.0001 |
| U3 | Group *F*_1.15_ = 12.32, *p* = 0.032  Amplitude *F*_4,60_ = 46.67, *p* < 0.0001 | Amplitude x Group *F*_4,60_ = 36.42, *p* < 0.0001 |
| D3 | Group *F*_1,17_ = 31.16, *p* < 0.0001  Amplitude *F*_4,68_ = 58.17 *p* < 0.001 | Amplitude x Group *F*_4,68_ = 26.51, *p* < 0.0001 |
| L4 | Group *F*_1,26_ = 15.79, *p* = 0.0005  Amplitude *F*_4,104_ = 61.26, *p* < 0.0001 | Amplitude x Group *F*_4,104_ = 3.10, *p =* 0.0188 |
| L5 | Group *F*_1,26_ = 3.15, *p* =0.0875  Amplitude *F*_4,104_ = 71.95, *p* < 0.0001 | Amplitude x Group *F*_4,104_ = 0.46, *p* = 0.7622 |
|  | | |
| Response metric: Excitatory area under the curve (EAUC) in the onset response analysis window from 5-50 ms from stimulus onset**.** | | |
| **Layer** | **Main terms** | **Interaction terms** |
| L2 | Group *F*_1,14_ = 5.67, *p* = 0.0320  Amplitude *F*_4,56_ = 5.67, *p* < 0.0001 | Amplitude x Group *F*_4,56_ = 7.58, *p* = 0.0001 |
| U3 | Group *F*_1,15_ = 18.28, *p* = 0.0007  Amplitude *F*_4,60_ = 22.14, *p* < 0.0001 | Amplitude x Group *F*_4,60_ = 18.22, *p<*= 0.0001 |
| D3 | Group *F*_1,17_ = 14.24, *p* = 0.0015  Amplitude *F*_4,68_= 31.01, *p* < 0.0001 | Amplitude x Group *F*_4,68_ = 9.05, *p<* 0.0001 |
| L4 | Group *F*_1,27_ = 13.69, *p* = 0.001  Amplitude *F*_4,108_ = 21.20, *p* < 0.0001 | Amplitude x Group *F*_4,108_ = 2.91, *p =*  0.0249 |
| L5 | Group *F*_1,26_ = 2.56, *p* = 0.1216  Amplitude *F*_4,104_ = 32.89, *p* < 0.0001 | Amplitude x Group *F*_4,104_ = 1.14, *p* = 0.3405 |
|  | | |
| Response metric: Latency to PFR in the onset response analysis window from 5-50 ms from stimulus onset**.** | | |
| **Layer** | **Main terms** | **Interaction terms** |
| L2 | Group *F*_1,14_ = 1.22, *p* = 0.2884  Amplitude *F*_4,56_ = 4, *p* = 0.0064 | Amplitude x Group *F*_4,56_ = 1.53, *p =* 0.2057 |
| U3 | Group *F*_1,15_ = 0.67, *p* = 0.4258  Amplitude *F*_4,60_ = 4.83, *p* = 0.0019 | Amplitude x Group *F*_4,60_ = 0.39, *p* = 0.8169 |
| D3 | Group *F*_1,17_ = 1.23, *p* = 0.2820  Amplitude *F*_4,68_ = 7.95 *p* < 0.0001 | Amplitude x Group *F*_4,68_ = 1.05, *p* = 0.3877 |
| L4 | Group *F*_1,27_ = 0.10, *p* = 0.7493  Amplitude *F*_4,108_ = 12.83, *p* < 0.0001 | Amplitude x Group *F*_4,108_ = 0.66, *p =* 0.6242 |
| L5 | Group *F*_1,26_ = 0.01, *p* =0.9332  Amplitude *F* _4,104_ = 16.03, *p* < 0.0001 | Amplitude x Group *F*_4,104_ = 2.17, *p* = 0.0775 |
|  | | |
| Response metric: Half-peak width (HPW) in the onset response analysis window from 5-50 ms from stimulus onset**.** | | |
| **Layer** | **Main terms** | **Interaction terms** |
| L2 | Group *F*_1,14_ = 9.52, *p* = 0.0081  Amplitude *F*_4,56_ = 0.31, *p* = 0.8683 | Amplitude x Group *F*_4,56_ = 2.55, *p =* 0.0493 |
| U3 | Group *F*_1,15_ = 15.14, *p* = 0.0014  Amplitude *F*_4,60_ = 0.45, *p* = 0.7693 | Amplitude x Group *F*_4,60_ = 15.14, *p* = 0.1733 |
| D3 | Group *F*_1,17_ = 25.91, *p* < 0.0001  Amplitude *F*_4,68_ = 1.22 *p* = 0.3093 | Amplitude x Group *F*_4,68_ = 1.35, *p* = 0.2606 |
| L4 | Group *F*_4,108_ = 0.54, *p* = 0.7088  Amplitude *F*_4,108_ = 0.54, *p =* 0.7088 | Amplitude x Group *F*_4,108_ = 1.29, *p =* 0.2803 |
| L5 | Group *F*_1,26_ = 0.87, *p* =0.3587  Amplitude *F*_4,104_ = 0.06, *p* = 0.9927 | Amplitude x Group *F*_4,104_ = 1.30, *p* = 0.2753 |
