# Supplementary material for: Cortical Hypoexcitation Defines Neuronal Responses in the Immediate Aftermath of Traumatic Brain Injury
Source: PLoS One. 2013 May 7;8(5):e63454. doi: 10.1371/journal.pone.0063454 (PMC3646737; doi:10.1371/journal.pone.0063454)
Supplement: Table S2 — Results of Two-way repeated measures ANOVA statistical analysis of peak firing rate, excitatory area under the curve, latency to peak firing rate and half-peak width in clusters responsive to the object contact stimulus from 5–50 ms from stimulus onset (related to Figures 4B and 5B ). The Table lists F statistics and degrees of freedom for both significant and non-significant factors for main and interaction terms. (DOCX) [file pone.0063454.s005.docx]

**Supplementary Data**

**Table S2. Results of Two-way repeated measures ANOVA statistical analysis of firing rate (PFR), excitatory area under the curve (EAUC), latency to PFR and half-peak width (HPW) in clusters responsive to the object contact stimulus from 5-50ms from stimulus onset (related to Figures 4B and 5B).** The Table lists F statistics and degrees of freedom for both significant and non-significant factors for main and interaction terms.

| Response metric: Peak Excitatory Firing Rate (PFR_on_) in the onset response analysis window from 5-50 ms from stimulus onset**.** | | |
| --- | --- | --- |
| **Layer** | **Main terms** | **Interaction terms** |
| L2 | Group *F*_1,14_ = 4.86, *p* = 0.0447  Amplitude *F*_9,126_ = 3.06, *p* = 0.0024 | Amplitude x Group *F*_9,126_ = 2.68, *p* = 0.0069 |
| U3 | Group *F*_1,14_ = 16.19, *p* = 0.0013  Amplitude *F*_9,126_ = 4.61 , *p* < 0.0001 | Amplitude x Group *F*_9,126_ = 1.73, *p* = 0.0885 |
| D3 | Group *F*_1,14_ = 27.43, *p* = 0.0001  Amplitude *F*_9,126_ = 10.09 *p* < 0.0001 | Amplitude x Group *F*_9,126_ = 2.52, *p* = 0.0110 |
| L4 | Group *F*_1,29_ = 10.28, *p* = 0.0033  Amplitude *F*_9,261_ = 18.42, *p* < 0.0001 | Amplitude x Group *F*_9,261_ = 0.67, *p =* 0.7315 |
| L5 | Group *F*_1,26_ = 4.05, *p* =0.0546  Amplitude *F*_9,234_ = 22.72, *p* < 0.0001 | Amplitude x Group *F*_9,234_ = 1.82, *p* = 0.0650 |
|  | | |
| Response metric: Excitatory area under the curve (EAUC) in the onset response analysis window from 5-50 ms from stimulus onset**.** | | |
| **Layer** | **Main terms** | **Interaction terms** |
| L2 | Group *F*_1,14_ = 5.55, *p* = 0.0336  Amplitude *F*_9,126_ = 6.10, *p* < 0.0001 | Amplitude x Group *F*_9,126_ = 5.64, *p* < 0.0001 |
| U3 | Group *F*_1,14_ = 21.86, *p* = 0.0004  Amplitude *F*_9,126_ = 15.23, *p* < 0.0001 | Amplitude x Group *F*_9,126_ = 11.94, *p* < 0.0001 |
| D3 | Group *F*_1,14_ = 31.91, *p* < 0.0001  Amplitude *F*_9,126_ = 25.48, *p* < 0.0001 | Amplitude x Group *F*_9,126_ = 12.59, *p<* 0.0001 |
| L4 | Group *F*_9,270_ = 33.06, *p* < 0.0001  Amplitude *F*_9,270_ = 33.06, *p* < 0.0001 | Amplitude x Group *F*_9,270_ = 4.21, *p* < 0.0001 |
| L5 | Group *F*_1,26_ = 3.00, *p* = 0.0951  Amplitude *F*_9,234_ = 53.04, *p* < 0.0001 | Amplitude x Group *F*_9,234_ = 1.47, *p* = 0.1605 |
|  | | |
| Response metric: Latency to PFR in the onset response analysis window from 5-50 ms from stimulus onset**.** | | |
| **Layer** | **Main terms** | **Interaction terms** |
| L2 | Group *F*_1,14_ = 0.07, *p* = 0.8011  Amplitude *F*_9,126_ = 1.22, *p* = 0.2822 | Amplitude x Group *F*_9,126_ = 0.93, *p =* 0.5023 |
| U3 | Group *F*_1,14_ = 3.09, *p* = 0.1005  Amplitude *F*_9,126_ = 2.09, *p* = 0.0354 | Amplitude x Group *F*_9,126_ = 1.40, *p* = 0.1941 |
| D3 | Group *F*_1,14_ = 0.14, *p* = 0.7095  Amplitude *F*_9,126_ = 3.97 *p* = 0.0002 | Amplitude x Group *F*_9,126_ = 0.94, *p* = 0.4936 |
| L4 | Group *F*_1,30_ = 0.43, *p* = 0.5164  Amplitude *F*_9,270_ = 24.69, *p* < 0.0001 | Amplitude x Group *F*_9,270_ = 0.80, *p =* 0.6146 |
| L5 | Group *F*_1,32_ = 0.82, *p* =0.3733  Amplitude *F*_9,288_ = 18.63, *p* < 0.0001 | Amplitude x Group *F*_9,288_ = 2.78, *p* = 0.0039 |
|  | | |
| Response metric: Half-peak width (HPW) in the onset response analysis window from 5-50 ms from stimulus onset**.** | | |
| **Layer** | **Main terms** | **Interaction terms** |
| L2 | Group *F*_1,14_ = 11.58, *p* = 0043  Amplitude *F*_9,126_ = 1.18, *p* = 0.3162 | Amplitude x Group *F*_9,126_ = 0.57, *p =* 0.8232 |
| U3 | Group *F*_1,14_ = 32.48, *p* < 0.0001  Amplitude *F*_9,126_ = 1.02, *p* = 0.4261 | Amplitude x Group *F*_9,126_ = 1.08, *p* = 0.3800 |
| D3 | Group *F*_1,14_ = 6.53, *p* = 0.0229  Amplitude *F*_9,126_ = 1.70 *p* = 0.0952 | Amplitude x Group *F*_9,126_ = 0.74, *p* = 0.6749 |
| L4 | Group *F*_1,30_ = 6.36, *p* = 0.0172  Amplitude *F* _9,270_ = 5.42, *p* < 0.0001 | Amplitude x Group *F*_9,270_ = 0.73, *p =* 0.6874 |
| L5 | Group *F*_1,26_ = 2.56, *p* =0.1217  Amplitude *F*_9,234_ = 5.45, *p* < 0.0001 | Amplitude x Group *F*_9,234_ = 0.40, *p* = 0.9345 |
