# Supplementary material for: Cortical Hypoexcitation Defines Neuronal Responses in the Immediate Aftermath of Traumatic Brain Injury
Source: PLoS One. 2013 May 7;8(5):e63454. doi: 10.1371/journal.pone.0063454 (PMC3646737; doi:10.1371/journal.pone.0063454)
Supplement: Table S3 — Results of Two-way repeated measures ANOVA statistical analysis of peak firing rate, excitatory area under the curve, latency to peak firing rate and half-peak width in clusters responsive to the rough surface discrimination whisker motion stimulus from 5–30 ms from stimulus onset (related to Figures 4C and 5C ). The Table lists F statistics and degrees of freedom for both significant and non-significant factors for main and interaction terms. (DOCX) [file pone.0063454.s006.docx]

**Supplementary Data**

**Table S3. Results of Two-way repeated measures ANOVA statistical analysis of firing rate (PFR), excitatory area under the curve (EAUC), latency to PFR and half-peak width (HPW) in clusters responsive to the rough surface discrimination whisker motion stimulus from 5-30ms from stimulus onset (viz. to Figures 4C and 5C).** The Table lists F statistics and degrees of freedom for both significant and non-significant factors for main and interaction terms.

| Response metric: Peak Excitatory Firing Rate (PFR_on_) in the onset response analysis window from 5-30 ms from stimulus onset**.** | | |
| --- | --- | --- |
| **Layer** | **Main terms** | **Interaction terms** |
| L2 | Group *F*_1,12_ = 8.55, *p* = 0.0127  Amplitude *F*_9,108_ = 2.93, *p* = 0.0038 | Amplitude x Group *F*_9,108_ = 1.74, *p* = 0.0884 |
| U3 | Group *F*_1,12_ = 26.94, *p* = 0.0002  Amplitude *F*_9,108_ = 9.83 , *p* < 0.0001 | Amplitude x Group *F*_9,108_ = 3.51, *p* = 0.0008 |
| D3 | Group *F*_1,15_ = 24.63, *p* = 0.0002  Amplitude *F*_9,135_ = 23.60 *p* < 0.0001 | Amplitude x Group *F*_9,135_ = 0.79, *p* = 0.6265 |
| L4 | Group *F*_1,28_ = 9.27, *p* = 0.0050  Amplitude *F*_9,252_ = 18.76, *p* < 0.0001 | Amplitude x Group *F*_9,252_ = 2.56, *p =* 0.0078 |
| L5 | Group *F*_1,25_ = 4.20, *p* = 0.0512  Amplitude *F*_9,225_ = 18.86, *p* < 0.0001 | Amplitude x Group *F*_9,225_ = 3.36, *p =* 0.0007 |
|  | | |
| Response metric: Excitatory area under the curve (EAUC) in the onset response analysis window from 5-30 ms from stimulus onset**.** | | |
| **Layer** | **Main terms** | **Interaction terms** |
| L2 | Group *F*_1,12_ = 12.01, *p* = 0.0047  Amplitude *F*_9,108_ = 3.01, *p* = 0.0031 | Amplitude x Group *F*_9,108_ = 2.37, *p* = 0.0173 |
| U3 | Group *F*_1,12_ = 46.34, *p* < 0.0001  Amplitude *F*_9,108_ = 12.03, *p* < 0.0001 | Amplitude x Group *F*_9,108_ = 8.09, *p* < 0.0001 |
| D3 | Group *F*_1,15_ = 29.75, *p* < 0.0001  Amplitude *F*_9,135_ = 35.38, *p* < 0.0001 | Amplitude x Group *F*_9,135_ = 7.40, *p<* 0.0001 |
| L4 | Group *F*_1,28_ = 19.86, *p* = 0.0001  Amplitude *F*_9,252_ = 7.98, *p* = 0.0001 | Amplitude x Group *F*_9,252_ = 2.08, *p =* 0.0315 |
| L5 | Group *F*_1,25_ = 6.08, *p* = 0.0209  Amplitude *F*_9,225_ = 8.86, *p* < 0.0001 | Amplitude x Group *F*_9,225_ = 2.96, *p* = 0.0024 |
|  | | |
| Response metric: Latency to PFR (LTP) in the onset response analysis window from 5-30 ms from stimulus onset**.** | | |
| **Layer** | **Main terms** | **Interaction terms** |
| L2 | Group *F*_1,12_ = 0.71, *p* = 0.4154  Amplitude *F*_9,108_ = 2.54, *p* = 0.0111 | Amplitude x Group *F*_9,108_ = 1.36, *p =* 0.2170 |
| U3 | Group *F*_1,12_ = 1.29, *p* = 0.2774  Amplitude *F*_9,108_ = 1.03, *p* = 0.4214 | Amplitude x Group *F*_9,108_ = 1.60, *p* = 0.1252 |
| D3 | Group *F*_1,15_ = 0.67, *p* = 0.4274  Amplitude *F*_9,135_ = 1.87 *p* = 0.0610 | Amplitude x Group *F*_9,135_ = 0.56, *p* = 0.8285 |
| L4 | Group *F*_1,30_ = 1.36, *p* = 0.2519  Amplitude *F*_9,270_ = 17.14, *p* < 0.0001 | Amplitude x Group *F*_9,270_ = 1.13, *p =* 0.3403 |
| L5 | Group *F*_1,25_ = 0.64, *p* =0.4309  Amplitude *F*_9,225_ = 2.69, *p* < 0.0054 | Amplitude x Group *F*_9,225_ = 1.62, *p* = 0.1103 |
|  | | |
| Response metric: Half-peak width (HPW) in the onset response analysis window from 5-30 ms from stimulus onset**.** | | |
| **Layer** | **Main terms** | **Interaction terms** |
| L2 | Group *F*_1,12_ = 8.10, *p* = 0.0147  Amplitude *F*_9,108_ = 2.01, *p* = 0.0445 | Amplitude x Group *F*_9,108_ = 1.17, *p =* 0.3217 |
| U3 | Group *F*_1,12_ = 68.03, *p* < 0.0001  Amplitude *F* _9,108_ = 2.19, *p* = 0.0283 | Amplitude x Group *F*_9,108_ = 1.54, *p* = 0.1441 |
| D3 | Group *F*_1,15_ = 2.02, *p* = 0.1761  Amplitude *F*_9,135_ = 0.62 *p* = 0.7765 | Amplitude x Group *F*_9,135_ = 0.69, *p* = 0.7188 |
| L4 | Group *F*_1,28_ = 4.59, *p* = 0.0410  Amplitude *F*_9,252_ = 0.91, *p* = 0.5142 | Amplitude x Group *F*_9,252_ = 1.16, *p =* 0.3212 |
| L5 | Group *F*_1,25_ = 2.56, *p* =0.1224  Amplitude *F*_9,225_ = 1.03, *p* = 0.4158 | Amplitude x Group *F*_9,225_ = 1.27, *p* = 0.2543 |
